# Death from Chloroform at the Bellevue Hospital, New York

**Published:** 1862-04

**Authors:** 


Death from Chloroform at the Bellevue Hospital, New York.—The occurrence of a death from chloroform in a metropolitan hospital, although the verdict of the coroner's jury exculpates the house-surgeon who administered it, suggests the inquiry whether the responsibility really devolving upon those who use so dangerous an agent can be thus readily shifted, and whether, in the light of present experience, it is not censurable, to use no stronger term, to expose patients, by the employment of one anæsthetic, to the risk of losing their lives, when in another we possess the means of securing an insensibility which is entirely satisfactory, and at the same time perfectly safe.

In the present instance, chloroform was given to a young woman, admitted to the hospital on the 3d ult., for the purpose of manipulating a recent injury of the shoulder joint; but a very small quantity was used, and that with every precaution, and the patient was not brought profoundly under its influence. She died before the examination was completed. We hear nothing against the quality of the article used; on the contrary it is said to have been perfectly satisfactory. There was nothing unusual in the condition of the patient, the accident, or the circumstances of inhalation; it was a common case of death from chloroform, affording still another illustration of what all surgeons know, though some of them are reluctant to acknowledge the fact (witness the silence of the New York medical journals with regard to this case), that no care taken, and no purity of the chloroform used, can avert the fatal shafts of this treacherous anæsthetic. It is very true, and fortunately so, that an event of this nature is of comparatively rare occurrence; that it happens for the first time in any particular hospital, can not, however, be claimed as a credit by its surgeons; they have only their good fortune to thank that so long a period of immunity in its use has been permitted them.

We learn that by a vote of the Surgeons of the New York Hospital, chloroform has not been used in that institution for twelve or fifteen years. A resolution formerly offered at the Bellevue Hospital that it should be administered only under the supervision of the attending physicians and surgeons, was not adopted. Such a recommendation now forms a part of the finding of the inquest. If the testimony of Dr. Willard Parker and Dr. James R. Wood in favor of ether is not to be heeded, it is to be hoped the suggestion of the jury will at least be listened to, and that the use of chloroform will henceforth be restricted to as few persons as possible. It is fortunate, perhaps, that the young gentlemen of the medical class of the Bellevue Hospital College, before being scattered to their homes, have had their attention forcibly called to the dangers of an agent which many of them, undoubtedly, have felt to be so innoccuous in its nature. We are glad, too, if such occurrences, we can hardly call them accidents, must happen, that this one has preceded the vote about to be taken by the New York Academy of Medicine on the conclusions of Dr. Barker's paper on the use of anæsthetics in midwifery. It can not but make every member feel deeply the responsibility attaching itself to a vote which endorses the superior merits of chloroform for any purpose whatever. Only the other day a correspondent of an English medical journal, writing from Paris, spoke of the hesitation with which chloroform was there given, and of the cries of the half-chloroformed patients undergoing operations by hospital surgeons who do not dare to withhold an anæsthetic, and yet give it with fear and trembling. It seems to us, if facts accumulate as they have heretofore, and men's minds remain open to conviction, and unbiassed by prejudice or partisan feeling, that the responsibility attaching itself to the exhibition of chloroform will soon be found here, as it bids fair to be in Paris, and (we are told by Dr. Hay, of this city, who has just returned from there) in Vienna also, greater than most surgeons will feel inclined to add to that of an operation.

Our readers will have anticipated us when we say that none of these apprehensions accompany the use of sulphuric ether. It is very easy to heap abuse upon the odor, or the bulk of this anæsthetic agent, or upon the zeal with which Boston defends its claims, and to assert that deaths have been caused by its inhalation. It is not so easy to point them out and prove them such. The burthen of proof rests with the friends and defenders of chloroform. That they have done this we are not aware. It is true that certain cases contained in the ether report of the Medical Improvement Society of this city have been quoted in a very general way against the committee, and one of their conclusions questioned; but no one, we believe, has undertaken to maintain, or show that in any particular instance a fatal result was really and unquestionably due to the inhalation of pure sulphuric ether. The extent of objection has been that it is not made perfectly clear in certain cases that death *may* not have resulted from this cause. But without regard to the views of that committee, or the Society it represents, of the safety and entire efficiency of sulphuric ether there can be no doubt. Considerations of humanity, therefore, ask for a fair and candid trial of its claims to produce perfect anæsthesia, with a degree of security far superior to any other anæsthetic yet discovered; and we feel perfectly assured that any one who once becomes familiar with its use will learn to place implicit confidence in the reliability of its properties.

The above from the Boston Medical and Surgical Journal presents us with another case of death from chloroform, in which "nobody is to blame." It has long been a matter of wonder to us, that there has not been more hesitation and reluctance in the administration of chloroform; the record of death by this agent comes to us every month, and yet many seem determined to use it, till *they* have a "case."

Its employment is of course more admissible when the patient at best stands between life and death,—in grave operations. We have always regarded its use, in cases where the life of the patient was not in jeopardy by the operation, of exceedingly doubtful propriety, and more so, because the great majority of deaths were in cases where very slight operations were to be performed, and more also, no one can tell when or where the fatal shaft will strike. It is true that great care will avert many an accident, but often, as in the present case, the greatest care in every particular will fail to prevent a fatal result. Every operator, who places his patient under the influence of chloroform, especially for a minor operation, puts life in jeopardy.

In ordinary dental practice, the use of chloroform is little short of criminal; it is to say the least unwarranted and rash. We can not understand the propriety of a dentist throwing his patient into the jaws of death, simply to extract a tooth without pain. We have never seen chloroform administered for such a purpose, without feeling a kind of horror. We would rejoice at some enactment that would forbid its use, save in those cases where life is in danger by the shock of an operation.

It is pleasing to know that there is a strong and growing opposition to the indiscriminate use of chloroform.—Ed. Reg.

